# Prenatal nicotine exposure during pregnancy results in adverse neurodevelopmental alterations and neurobehavioral deficits

**DOI:** 10.3389/adar.2023.11628

**Published:** 2023-08-11

**Authors:** Alicia C. Wells, Shahrdad Lotfipour

**Affiliations:** ^1^ School of Medicine, University of California, Irvine, Irvine, CA, United States; ^2^ Department of Emergency Medicine, Pharmaceutical Sciences, Pathology and Laboratory Medicine, University of California, Irvine, Irvine, CA, United States

**Keywords:** tobacco, neonatal exposure, teratogen, e-cigarettes, maternal smoking

## Abstract

Maternal tobacco use and nicotine exposure during pregnancy have been associated with adverse birth outcomes in infants and can lead to preventable pregnancy complications. Exposure to nicotine and other compounds in tobacco and electronic cigarettes (e-cigarettes) has been shown to increases the risk of miscarriage, prematurity, stillbirth, low birth weight, perinatal morbidity, and sudden infant death syndrome (SIDS). Additionally, recent data provided by clinical and pre-clinical research demonstrates that nicotine exposure during pregnancy may heighten the risk for adverse neurodevelopmental disorders such as Attention-Deficit Hyperactivity (ADHD), anxiety, and depression along with altering the infants underlying brain circuitry, response to neurotransmitters, and brain volume. In the United States, one in 14 women (7.2%) reported to have smoked cigarettes during their pregnancy with the global prevalence of smoking during pregnancy estimated to be 1.7%. Approximately 1.1% of women in the United States also reported to have used e-cigarettes during the last 3 months of pregnancy. Due to the large percentage of women utilizing nicotine products during pregnancy in the United States and globally, this review seeks to centralize pre-clinical and clinical studies focused on the neurobehavioral and neurodevelopmental complications associated with prenatal nicotine exposure (PNE) such as alterations to the hypothalamic-pituitary-adrenal (HPA) axis and brain regions such as the prefrontal cortex (PFC), ventral tegmental area (VTA), nucleus accumbens (NA), hippocampus, and caudate as well as changes to nAChR and cholinergic receptor signaling, long-term drug seeking behavior following PNE, and other related developmental disorders. Current literature analyzing the association between PNE and the risk for offspring developing schizophrenia, attention-deficit hyperactivity disorder (ADHD), autism spectrum disorder (ASD), anxiety, and obesity will also be discussed.

## Introduction

**FIGURE 1:**
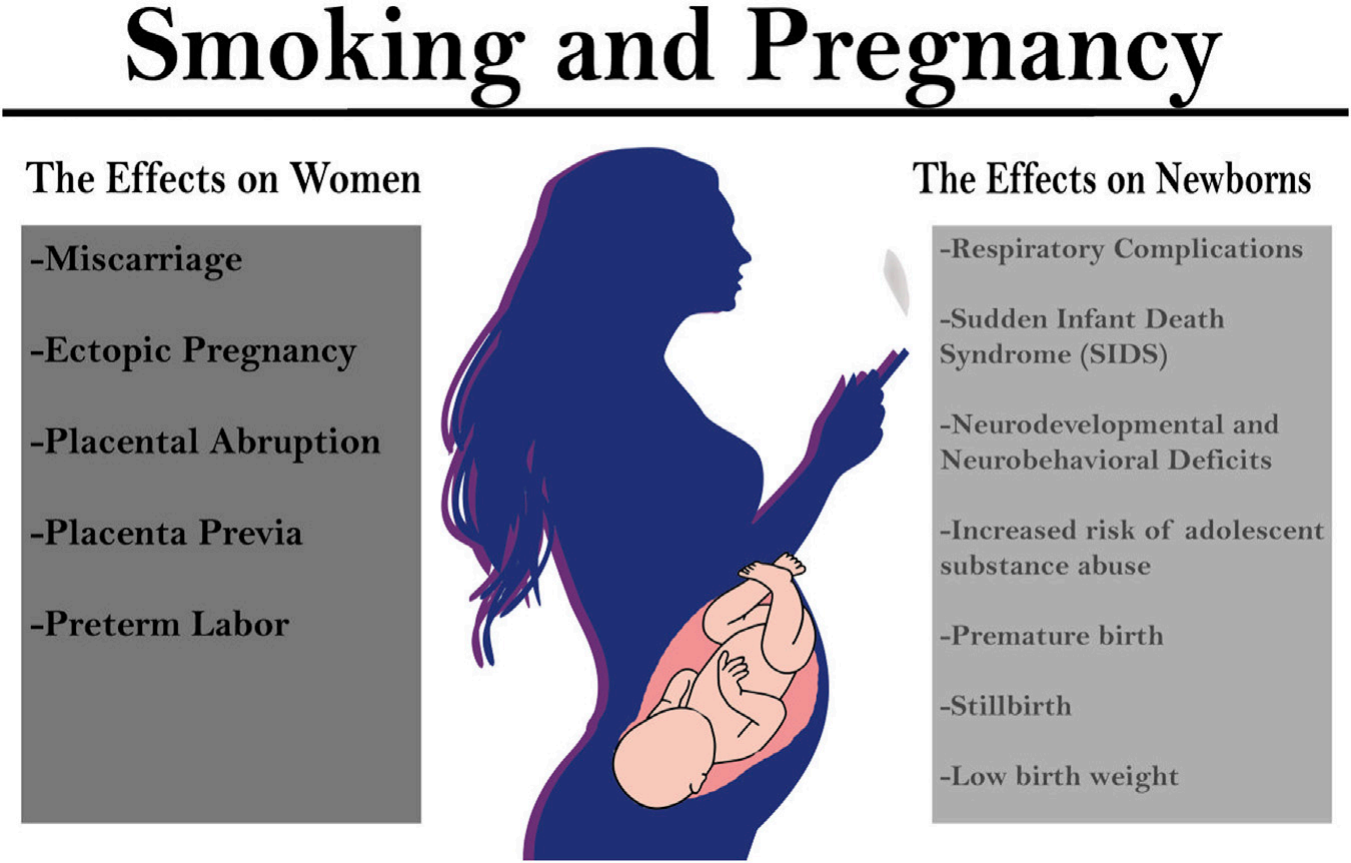
Consequences of smoking during pregnancy on women and their offspring. Research has indicated that smoking during pregnancy has both immediate and long-term consequences for women and their offspring. Pregnant women that smoke tobacco cigarettes are at a higher risk for miscarriage, ectopic pregnancy, placental abruption, placenta previa, and preterm labor. Offspring prenatal nicotine exposure (PNE) are at a higher risk for premature birth, stillbirth, and low birth weight as well as sudden infant death syndrome (SIDS), and respiratory complications. Offspring exposed to PNE are also at an increased risk for substance abuse and developing neurodevelopmental and neurobehavioral deficits.

Smoking tobacco is the leading preventable cause of death and disease in the U.S. leading to approximately one in every five deaths [1]. Despite there being an established risk between prenatal maternal smoking and adverse outcomes, as of 2016 one in fourteen women (7.2%) in the United States reported to have smoked cigarettes during their pregnancy with the global prevalence of smoking during pregnancy estimated to be 1.7% [2, 3]. Approximately 1.1% of women in the United States also reported to have used electronic cigarettes (e-cigarettes) during the last 3 months of pregnancy [4]. Maternal smoking during pregnancy is associated with immediate short-term consequences, like placental abruption, preterm birth, low birth weight and fetal growth restriction, stillbirth, and sudden infant death syndrome (Figure 1) [5–7]. Prenatal maternal smoking has also been associated with long-term adverse effects that extend into childhood and adolescence such as an increased risk for ADHD, obesity, schizophrenia, and adolescent substance abuse and dependence (Figure 1) [8–11]. Although the pathophysiological mechanisms of these consequences are poorly understood, changes to oxidative stress, mitochondrial function, placental apoptosis, and changes to brain volume, circuitry, and reward pathways are hypothesized as contributors [12–16].

Due to the large percentage of women utilizing nicotine products during pregnancy in the United States and globally, this review seeks to centralize the results of pre-clinical and clinical research focused on the neurobehavioral and neurodevelopmental complications associated with prenatal nicotine exposure (PNE). First, the pharmacology of nicotine, the primary psychoactive component of tobacco cigarettes, will be discussed including its role in neurotransmission, drug reward pathways, and nicotine dependence [17, 18]. The role of nicotinic acetylcholine receptors (nAChRs) in prenatal and postnatal neurodevelopment will then be discussed including their role in modulating the release of neurotransmitters across neurodevelopmental stages. We will then discuss how gestational exposure to nicotine activates or inhibits nAChRs and leads to deleterious changes in neurodevelopment such as decreased brain volume, upregulation of nAChRs, changes in signaling of the hypothalamic-pituitary-axis (HPA), and changes to brain regions such as the prefrontal cortex (PFC), ventral tegmental area (VTA), nucleus accumbens (NA), and caudate. The next sections will explore the impact of PNE on neurobehavioral outcomes such as attention-deficit hyperactivity disorder (ADHD), schizophrenia, autism spectrum disorder (ASD), and anxiety. The risk of obesity and adolescent substance abuse will also be discussed in relation to PNE. The Diagnostic and Statistical Manual of Mental Disorders, Fifth Edition (DSM-V) criteria for each disorder—the publication that provides the standard classification system and criteria for communicating about mental disorders among researchers, clinicians, and public health officials—will also be discussed for each disorder [19, 20]. Lastly, we will discuss nicotine replacement therapy (NRT) and electronic cigarettes (ECs), focusing on their current use during pregnancy and their posed risks to offspring.

## Pharmacology

**FIGURE 2:**
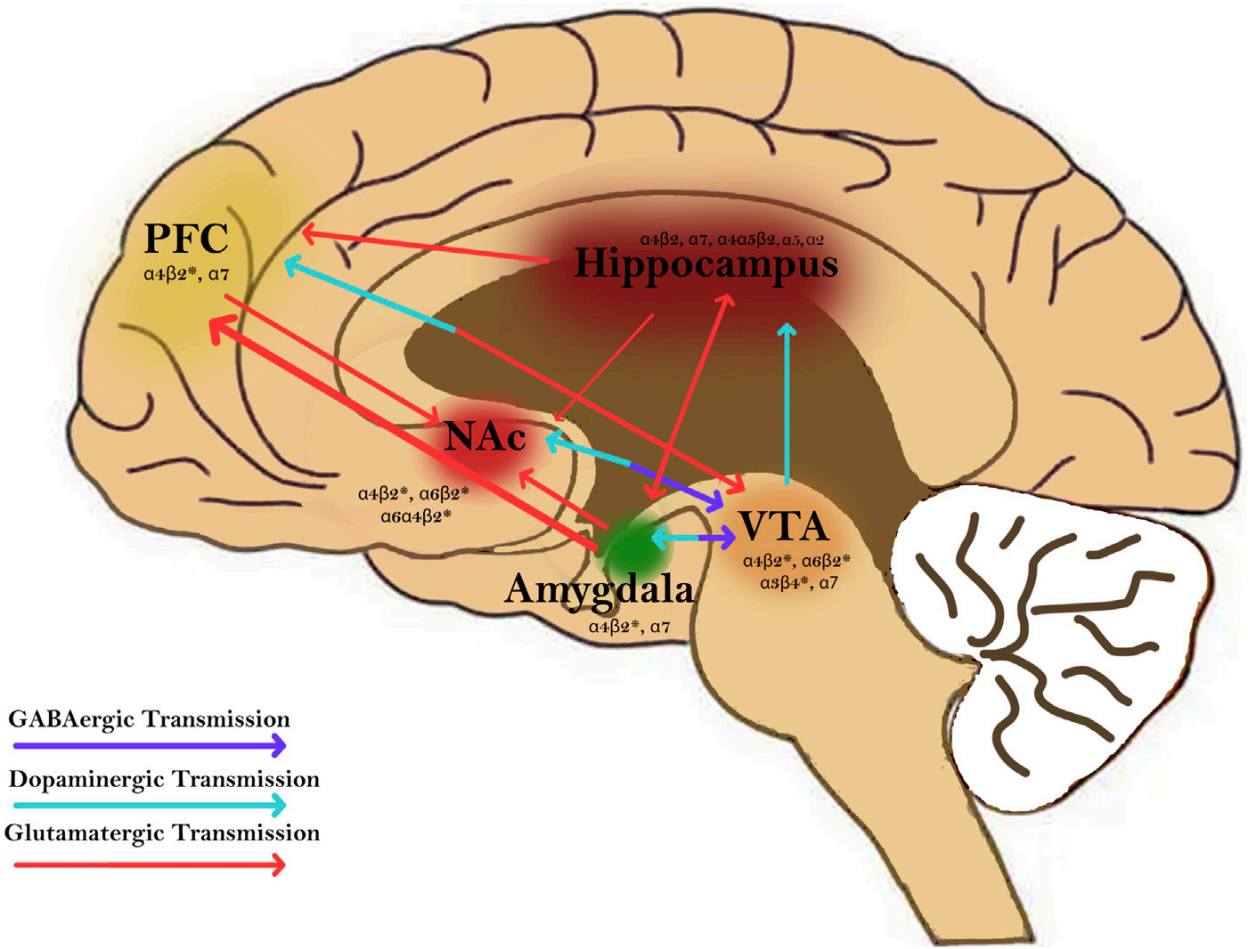
Nicotine activates nicotinic acetylcholine receptors (nAChRs) in brain regions and circuitries that are involved in mediating addition disorders. nAChRs are found in regions such as the prefrontal cortex (PFC), hippocampus, nucleus accumbens (NAc), amygdala, and ventral tegmental area (VTA). Nicotine modulates GABAergic (purple), dopaminergic (blue), and glutamatergic transmission (red) between these regions. Activation of nAChRs in these brain regions plays a major role in modulating synaptic plasticity and contributing to the rewarding and addictive effects of nicotine.

**FIGURE 3:**
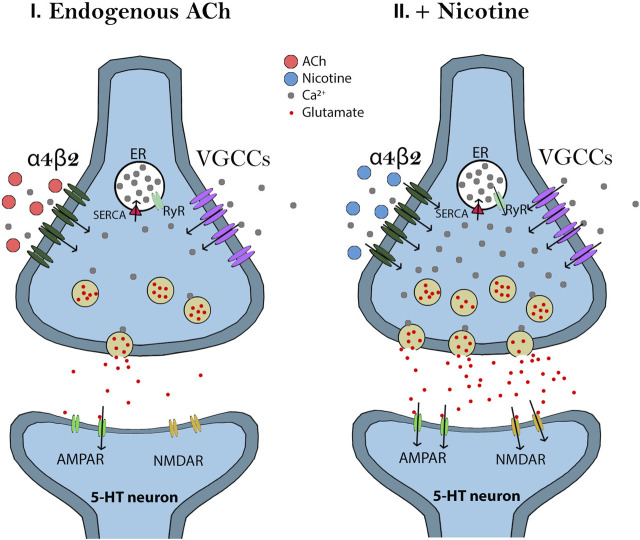
Effects of endogenous acetylcholine (Ach) and nicotine on dorsal raphe nucleus (DRN) 5-HT neurons. I) Endogenous Ach modulates the excitatory glutamatergic inputs to the DRN 5-HT neurons by activating α4β2 nAChRs at glutamate terminals. ACh is quickly metabolized by acetylcholinesterase which allows Ca2+ influx to be controlled and signaling at the terminal to be maintained at low levels. II) Nicotine also modulates the excitatory glutamatergic inputs to the DRN 5-HT neurons by activating α4β2 nAChRs at glutamate terminals. Since nicotine cannot be degraded, however, more α4β2 nAChRs are activated which leads to enhanced Ca2+ entry and voltage-gated calcium channel (VGCC) activation. The increased calcium influx will activate calcium-induced calcium release (CICR) from the endoplasmic reticulum (ER) through ryanodine receptor (RyR) signaling. Thus, nicotine generates a long-term potentiation of glutamate release.

Cigarette smoke is composed of a complex array of chemical constituents including products such as carboxylic acids, phenols, water, humectants, terpenoids, paraffin waxes, tobacco-specific nitrosamines, (TSNAs), and catechols [21]. When smoke is inhaled from a cigarette, nicotine is distilled from the tobacco and carried to the lungs where it is absorbed rapidly into the pulmonary venous circulation [17]. Nicotine then binds to neuronal nAChRs that mediate fast neurotransmission in the central and peripheral nervous system [22]. nAChRs are pentameric cation-selective ligand-gated ion channels composed of α and β subunits ( α 1–7, 9–10; β 1–4) that can signal the release of neurotransmitters such as dopamine, norepinephrine, acetylcholine, GABA, and glutamate involved in reward pathways, transmission at the neuromuscular junction, and cognition (Figures 2, 3) [18]. The main endogenous agonist of nAChRs is acetylcholine (ACh), but nicotine obtained endogenously from tobacco smoke or e-cigarettes can also serve as an agonist (Figure 2) [23].

**FIGURE 4:**
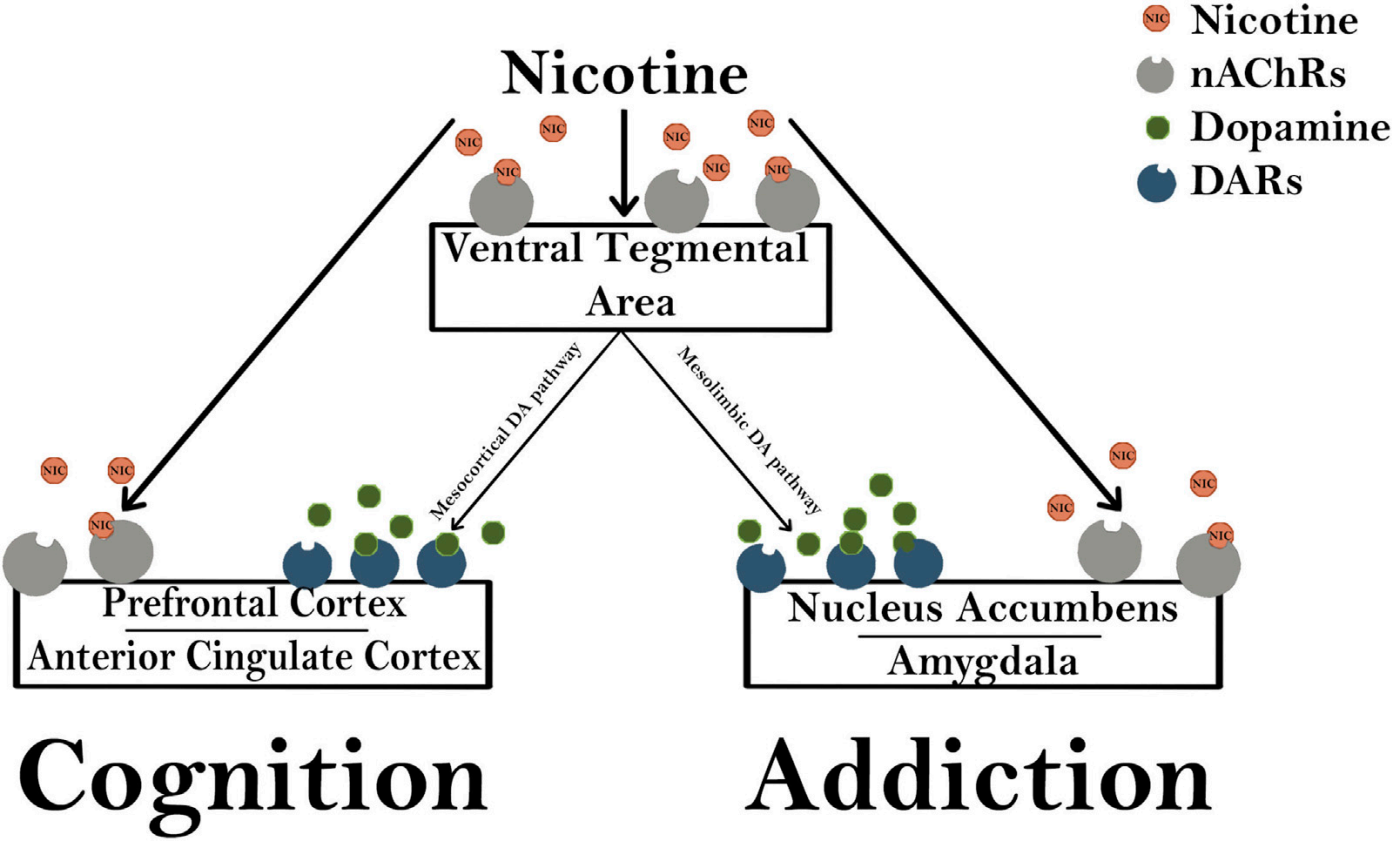
Nicotine modulates cognition and addiction pathways. Nicotinic acetylcholine receptors (nAChRs) in the ventral tegmental area (VTA) are activated by exogenous nicotine, stimulating both the mesocortical and mesolimbic dopaminergic (DA) pathways. The mesocortical DA pathway activates dopaminergic receptors (DARs) in the prefrontal cortex and anterior cingulate cortex that are important for the rewarding cognitive effects of nicotine. The mesolimbic pathway activates DARs in the nucleus accumbens and amygdala which are important for modulating changes in synaptic plasticity and long-term potentiation that are involved in addiction.

Brain imaging studies have demonstrated that nicotine acutely increases the activity in the visual system, PFC, and thalamus which are consistent with activation of the corticobasal ganglia-thalamic brain circuits [24]. Importantly, stimulation of nAChRs in dopaminergic neurons of the VTA of the midbrain and in the shell of the NA are critical in drug-induced reward pathways that are important for the perception of pleasure and reward (Figure 4) [18]. Thus, dopamine release causes a pleasurable experience that is critical for reinforcing the effects of tobacco and nicotine dependence [25].

While electronic cigarettes (e-cigarettes) do not produce combusted smoke, they do deliver an aerosol containing nicotine and other tobacco-related compounds [26]. The pharmacologic reason for continued use in the case of both tobacco cigarettes and e-cigarettes is due to nicotine’s ability to trigger the release of neurotransmitters producing rewarding psychoactive effects such as dopamine [18]. With repeated exposure to nicotine, tolerance develops and induces signs of physical dependence and withdrawal symptoms [18]. Due to the reinforcing effect that nicotine has on tobacco addiction behavior, pharmacologic interventions for tobacco cessation continue to be introduced including NRT’s in the form of gum, transdermal patches, oral inhalers, nasal sprays, and tablets [27]. Although NRT’s support tobacco abstinence, replacement therapies such as transdermal nicotine patches still have the potential to deliver between 5 mg and 22 mg of nicotine over a 24-hour period, resulting in plasma cotinine levels seen in heavy smokers. Thus, NRT’s still activates the same pharmacologic pathways as that of tobacco cigarettes and e-cigarettes.

## Prenatal and early postnatal development

### Nicotinic acetylcholine receptors (nAChRs)

From gestation to adulthood, nAChRs have been shown to play a functionally distinct role in the development of the brain through the modulation of neurotransmitter release across neurodevelopmental stages [28]. During the prenatal and postnatal periods of development, there are transient regional patterns of increased nAChR subunit expression that occur during critical periods of neural development involving synaptogenesis, neurogenesis, apoptosis, and migration [29].

Literature has documented that exposure to nicotine during fetal development has adverse effects on the developing offspring [29]. In addition to being activated by endogenous ligands during development, nAChRs can also be activated or inactivated by exogenous drugs such as nicotine (Figure 4) [30]. Following chronic PNE, high affinity nicotine binding in the neonatal and fetal brain is increased, providing evidence that nicotine reaches the fetal brain and upregulates nAChR receptors [16, 31–33]. Furthermore, activation or desensitization of nAChRs via PNE has been demonstrated to disrupt brain plasticity and programming into postnatal life [16, 34].

### Brain volume and characteristics

Gestational nicotine has also been shown to decrease the total number of cells in the whole brain and in certain neuronal subregions during the early fetal and neonatal period [16, 34]. Clinical studies have demonstrated an association between prenatal smoking and smaller total brain volume in regions such as the caudate nucleus, NA, frontal lobe, and cerebellum [14, 15]. The literature also reports that children exposed to prenatal smoking throughout pregnancy had thinning of the superior frontal cortex and precentral cortex which were associated with increased scores of affective problems and anxiety [15]. A cohort study of 2,704 children exposed to continued maternal smoking during pregnancy was also associated with lower regional brain volumes, surface area, and less gyrification at the age of 9–11 as compared with children not prenatally exposed to maternal smoking [35]. Similarly, a study using a mouse model found that PNE produced hyperactivity that was associated with selective decreases in cingulate cortical volume and radial thickness and a decrease in drug-induced dopamine release in the frontal cortex [36].

### Hypothalamic-pituitary-adrenal (HPA) axis

The fetal hypothalamic-pituitary-adrenal (HPA) axis is critical for mechanisms controlling the regulation of intrauterine homeostasis, the timely differentiation and maturation of vital organ systems such as the CNS, liver, and lungs, preparing the timing of fetal growth, and commencing parturition [37, 38]. Hormone activity in the HPA axis begins early in development between eight and 12 weeks of gestation and is regulated through interaction with the placenta [39]. Both animal and human studies have demonstrated that an adverse intrauterine environment can lead to HPA axis dysfunction [10, 40, 41]. PNE, has been shown to inhibit the functional development of the HPA axis and may increase one’s risk for intrauterine growth retardation (IUGR) [41]. A variety of clinical epidemiologic studies have demonstrated that the risk of chronic adult diseases, such as obesity, cardiovascular disease, diabetes, and depression are increased in IGUR offspring that were exposed to prenatal smoking or PNE [42, 43]. Clinical studies have also confirmed that prenatal glucocorticoid (GC) exposure not only sensitizes the HPA axis response to painful stress of a heel-stick in full-term infants, but also increases the stress reactivity of 10-year-old-children to a standard psychological stress test [44, 45]. One animal study demonstrated that PNE increases expression levels of cholesterol side-chain cleavage enzyme (P450scc) and adrenal steroid acute regulatory protein (StAR) in pregnant rats and reduces the levels of 11β-hydroxysteroid dehydrogenase-2 (11β-HSD-2) which is responsible for the inactivation of mother-derived GC [46]. Thus, PNE was found to alter the programming of the HPA axis and over-expose the fetus to maternal GC thereby increasing one’s risk for metabolic syndromes in adulthood [47].

### Epigenetics

Maternal smoking in pregnancy has also been associated with epigenetic modifications, including DNA methylation, that are implicated in deleterious changes to gene expression and processes related to growth and development [48, 49]. A meta-analysis analyzed the association between maternal smoking in pregnancy and newborn DNA methylation at over 450,000 CpG sites (cpGs) across 13 cohort studies (*n* = 6,685) [49]. They found that over 6,000 CpGs were differentially methylated in offspring exposed to maternal smoking including several genes that were critical in development [49]. Maternal smoking during pregnancy was found to significantly reduce the methylation of the gene PRDM8 (PR domain contain 8), which is a gene that’s tightly regulated in a spatiotemporal manner during neural development and is important for regulating the morphological transition in neocortical development [49–53]. The analysis also found DLGAP2 (discs large homolog-associated protein 2) to have many significant CpGs associated with maternal smoking during pregnancy [49].

DGLAP2 belongs to a gene family that encodes proteins that are involved in the molecular organization of synapses and in neuronal cell signaling and has been associated with progressive epilepsy with intellectual disabilities along with other CNS disorders such as ASD and schizophrenia [54–56]. Thus, maternal smoking during pregnancy is associated with epigenetic changes that may modulate the offspring’s risk for deleterious neurobehavioral and neurodevelopmental outcomes.

## Neurobehavioral and neurodevelopmental effects

### Attention-deficit hyperactivity disorder (ADHD)

Attention-Deficit Hyperactivity Disorder (ADHD) is a clinically heterogenous neurodevelopmental disorder characterized by hyperactivity, inattention, impulsiveness, and inattention that first presents during childhood [57]. The DSM-V characterizes ADHD as a persistent pattern of (A1) inattention (9 symptoms) and/or (A2) hyperactivity and impulsivity (9 symptoms) that interferes with function or development [20]. Symptoms must be present in two or more settings before the age of 12 and for at least 6 months and are required to impair occupational, social, or academic functioning [20, 58]. The diagnostic criteria include three ADHD subtypes: (1) Combined presentation of both hyperactivity and impulsivity meeting criterion A1 and A2 for at least 6 months, (2) Predominantly inattentive presentation meeting criterion A1 but not A2 for at least 6 months, and (3) Predominantly hyperactive/impulsive presentation meeting criterion A2 but not A1 for at least 6 months [20].

ADHD significantly impairs multiple aspects of life and has been associated with unsuccessful marriage, educational underachievement, unemployment, criminality, etc. [59, 60] Moreover, ADHD has been shown to have significant correlations with a wide range of comorbid psychiatric disorders, including defiant, antisocial personality disorder, self-harm, substance misuse, and affective disorders [61–63]. Globally, 5% of children and adolescents are estimated to have ADHD with a recent meta-analysis of twin studies estimating the heritability of ADHD to be 77%–88% [64, 65]. Although the etiology of ADHD is unclear, the high estimated heritability and population prevalence is consistent with ADHD being caused by multiple susceptibility genes in combination with other environmental risk factors [66].

#### Clinical research

A variety of clinical studies have sought to determine whether there is an association between PNE and one’s risk for ADHD [67–69]. A recent longitudinal cohort study (*n* = 100 cases, 100 controls) by Howell et al., for example, found prenatal maternal tobacco smoking to be a predictor of childhood ADHD and to be associated with shortened infant telomere length when controlling for maternal depression [8]. A large population-based study (*n* = 19,940) found that maternal smoking increased one’s risk for ADHD by 2.64 times (95% CI 1.45–4.80) and paternal smoking during pregnancy to increase one’s risk for ADHD by 1.17 times (95% CI 1.98–1.39) [70]. Another population-based study (*n* = 1,079) sought to control for recall bias related to self-reported data by examining the relationship between levels of maternal cotinine levels during pregnancy and risk of ADHD in offspring [69]. Their results indicated that offspring exposed to heavy nicotine exposure during gestation (cotinine level >50 ng/mL) had an increased risk (OR: 2.21; 95% CI 1.63–2.99) for ADHD as compared to offspring not exposed to maternal smoking during pregnancy when adjusting for demographic variables [69]. Another study (*n* = 3,216) analyzing maternal cotinine levels during pregnancy and one’s risk for ADHD found that maternal active smoking (cotinine level ≥ 11.49 ng/mL) during pregnancy was significantly associated with an increased risk of total difficulties (OR = 1.67) while maternal low- and high-passive smoking (cotinine level 0.22–0.51 ng/mL and 0.52–11.48 ng/mL respectively) increased the risk (OR = 1.11, 1.25, respectively) but was not statistically significant [67].

Multiple meta-analyses and systematic reviews have also addressed the association between PNE and ADHD [71–73]. One systematic review and meta-analysis of prospective cohort studies (*n* = 17,304), for example, found that maternal smoking during pregnancy was associated with an increased risk of ADHD (pooled RR = 1.58, 95% CI = [1.33, 1.88]) [73]. A meta-analysis of large population-based cohort studies (*n* = 3,076,173) also found that maternal smoking cessation during the first trimester was significantly associated with ADHD in offspring when adjusted for covariates (aOR 1.292, 95% CI 1.125–1.484) [72]. They also found that maternal passive smoking (paternal smoking) was significantly associated with offspring ADHD (OR aOR 1.106, 95% CI 0.968–1.264) but that the association became non-significant after adjusting for potential confounds [72]. Lastly, a meta-analysis of cohort and case-control studies (*n* = 50,044 cases, 2,998,059 total) found that smoking during pregnancy increased the risk of offspring ADHD (OR: 1.60; 95% CI:1.45–1.76) and that the risk of ADHD was greater in children whose mothers who smoked more than 10 or more cigarettes per day (OR: 1.75; 95% CI: 1.51–2.02) than for mothers that smoked fewer than 10 cigarettes per day (OR: 1.54; 95% CI: 1.40–1.70) [71].

#### Preclinical research

Since ADHD is a heterogenous disorder that results from different combinations of genetic and environmental factors, studying the disease in an animal model provides an invaluable resource for understanding the neurochemistry underlying specific aspects of ADHD and the relative risk that various exposures have on the development of ADHD [66]. Moreover, differences in behavior between the control and the animal model can be correlated with differences in their behavioral pharmacology and neurochemistry [66].

A list of criteria for an optimal animal model of ADHD was suggested and posits that the model should have impulsiveness be absent initially and gradually develop over time, should sustain attention-deficit behaviors only when stimuli are widely spaced in time, and should not display hyperactivity in a novel environment [74–77]. The different types of models that have been developed are used to study the neurochemical and pathological basis of ADHD [78]. At present, spontaneously hypertensive rats (SHR) are considered an appropriate animal model for ADHD due to their behavioral characteristics, genetic profile, and disruptions in their ability to metabolize and store dopamine [66]. Poor performers in the 5-choice serial reaction time (5-CSRT), dopamine transported knock-out (KO) mice, and nicotinic receptor KO mice have also been suggested as useful models for ADHD [77, 79].

In one study, the researchers sought to determine the impact that PNE on measures of attention and impulsivity in adult male Lister hooded rats using the 5-CSRT [80]. Exposing female rats to 0.06 mg/mL of a nicotine solution during pregnancy, they found that their offspring had lower birth weights and delayed sensorimotor development, grip strength, and righting reflex. In the 5-CSRT, adult offspring had an increased number of omissions errors and anticipatory responses, lower accuracy with evidence of delayed learning of the task demands, and more variable response times. They also found that PNE increased the expression of the D5 dopamine receptor in the striatum but did not influence exploratory locomotion, the delay-discounting test, or the expression of other dopamine-related genes (*DRD4*, *DAT1*, *NR4A2*, and *TH*) in either the PFC or the striatum [80]. These results indicate that when genetic variance and other environmental factors are controlled for, PNE can lead to cognitive changes that could contribute to components of the ADHD phenotype.

Another study in a mouse animal model found that PNE reduced the number of tyrosine hydroxylase (TH)-positive varicosities in the shell of the NA and in the mPFC [81]. They also found that PNE induced behavioral deficits in object-based attention, sensorimotor gating, and cliff avoidance in offspring. Moreover, they found that acute treatment with atomoxetine (3 mg/kg) or with methylphenidate (MPH) (1 mg/kg) where able to attenuate or partially attenuate the behavioral deficits respectively. These results indicate that PNE produces disruptions to the DAergic system that induce neurobehavioral abnormalities in mouse offspring and lead to ADHD-like behaviors [81].

Studies have also characterized a PNE mouse model that displays the full range of ADHD associated behavioral phenotypes including attention deficit and impulsive-like behavior and working memory deficits [82]. They found that when a single therapeutic equivalent dose of MPH (0.75 mg/kg) was administrated, the ADHD behavioral phenotype was attenuated. Recently, a study that utilized the PNE ADHD mouse model found that administration of MPH (1 mg/kg) restored the behavioral impairments and neuroplasticity changes in the AMPAR subunit composition and distribution while restoring the density and maturation of dendritic spines of the hippocampal pyramidal neurons (Figure 3) [83]. These results suggest that the ADHD PNE mouse model can recapitulate the key features of ADHD and may be used for further translational research in ADHD [82].

### Schizophrenia

Schizophrenia is a serious mental disorder characterized by delusions, hallucinations, disturbances in thought, perception, and behavior [84]. The symptoms of schizophrenia can generally be divided into negative symptoms, such as anhedonia, poverty of speech, and lack of motivation, and positive symptoms, such as delusions, hallucinations, and formal thought disorders [84]. According to the DSM-V, a schizophrenia diagnosis requires that an individual exhibits two of five main symptoms including hallucinations, delusions, disorganized or incoherent speaking, disorganized or speaking, and disorganized or unusual movements and negative symptoms [20]. The key symptoms must last for at least 1 month and must negatively impacting one’s quality of life and functioning [20].

The prevalence of schizophrenia is between 0.6% and 1.9% in the United States with over 21 million people afflicted worldwide [85, 86]. Due to its complexity, schizophrenia’s pathophysiology and etiology is not fully understood, but is believed to result from a complex interplay of genetic and environmental risk factors such as childhood trauma, family history, social isolation, birthing complications, severe maternal malnutrition, and cannabis use [87, 88]. According to family, twin, and adoption studies addressing one’s risk for developing schizophrenia, genetic factors play a major role, with its heritability estimated to be between 60% and 85% [89]. Additionally, pre-molecular and molecular genetic studies have demonstrated that several culprit genes are associated with an increased risk for developing schizophrenia with hundreds or even thousands of distinct genetic loci involved [90]. Specifically, genes encoding the dopamine receptor D2 are impacted, affecting pathways critical for attention, locomotion, goal-oriented behaviors, and reward [91]. Interestingly, patients with schizophrenia are also reported to have a smoking prevalence of up to 88%, nearly three times the rate of the general population and higher than rates of smoking in patients with other psychiatric illnesses [92]. Although there are several hypotheses for this association, more recent data has suggested that cigarette smoking may be reinforcing for patients due to its reduction of psychiatric symptoms [93].

Increasing evidence has supported a role between pre- and postnatal environmental risk factors that lead to neurodevelopment changes implicated in schizophrenia [94]. Although the literature has revealed some conflicting data on the role that PNE has in the development of schizophrenia, clinical and preclinical studies reveal that individuals may be at an increased risk for developing schizophrenia following PNE [9, 94].

#### Clinical research

In a population-based nested case-control study (*n* = 977), an association between higher maternal cotinine levels was associated with an increased odds of schizophrenia in offspring (OR = 3.41, 95% CI 1.86–6.24). Those categorically defined as heavy smokers (>50 ng/mL maternal cotinine) were found to have 38% increased odds of schizophrenia in offspring as compared to nonsmoking mothers when accounting for maternal age, SES, maternal or parental psychiatric disorders, and other covariates [94]. Another case-control study of one hundred patients with schizophrenia and 100 nonschizophrenia-matched subjects, contrarily, found that the amount of tobacco used during pregnancy did not differ between the mothers of cases and controls [95]. Indeed, they found that the amount of tobacco used was significantly lower in the mothers of patients with schizophrenia versus the mothers of non-schizophrenic subjects.

Another clinical study sought to determine whether prenatal maternal smoking was associated with changes to DNA methylation patterns in offspring that could mediate neurobehavioral changes such as schizophrenia [96]. Combing data from five prospective birth cohort studies, the researchers investigated the association between prenatal smoking exposure and offspring blood DNA methylation in 2821 adolescents and adults and identified 69 differentially methylated CpGs in 36 genomic regions of those exposed to maternal smoking. Further analysis provided evidence for a causal role of four maternal smoking related CpG sites in increasing the risk of schizophrenia and inflammatory bowel disease [96].

In a systematic review and meta-analysis, Hunter et al. pooled the data from four cohort studies and three-case control studies that reported data on the association between prenatal tobacco exposure and schizophrenia [9]. They found that those who had been exposed to maternal prenatal smoke had a 29% increased risk of developing schizophrenia (RR = 1.99; 95% CI = 1.10–3.61) [9]. In a letter written by Quinn et al. (2020), however, the authors argue that the meta-analysis failed to consider the role that familial confounding could have on the observed observation [97]. They highlight that observed associations between schizophrenia and maternal smoking during pregnancy may be the result of passive gene-environment correlation rather than a true effect of prenatal exposure to tobacco smoke [97].

#### Preclinical research

Preclinical studies have also sought to determine whether there is an association between PNE and one’s risk for developing schizophrenia while controlling for the potential confounds of clinical studies [98, 99]. Animal models of schizophrenia typically fit into four different induction categories including drug-induced, lesion or genetic manipulation, and developmental studies [100]. Neurodevelopmental models of schizophrenia in animals typically involve exposure of the neonate, either during gestation or the perinatal period, to adverse environmental insults such as maternal stress, hypoxia, malnutrition, or post-weaning social isolation that alter the brain development and cause behavioral deficits into adulthood [100–102]. Pharmacologic models of schizophrenia initially attempted to mimic the dysregulation of dopamine with hyperfunction of the mesolimbic dopamine system through amphetamine-induced psychosis [103, 104]. In recent years, however, increasing evidence supports dysfunction of the glutaminergic system as being the primary pathophysiological changes seen in schizophrenia [105]. Thus, non-competitive agonists that blockade the NMDA receptor, such as ketamine or phencyclidine (PCP), have been increasingly used in animal models of schizophrenia and have been shown to induce hyperlocomotion, social withdrawal, and impairment of both cognition and pre-pulse inhibition in rodents [100]. Neonatal lesions of the ventral hippocampus of the rat are also used as animal models of schizophrenia and cause developmental alterations to the PFC neuronal integrity, contributing to behavioral alterations [106]. Lastly, KO mice involving genes implicated in an increased risk for schizophrenia are used as rodent models.

An initial study in Wistar rats examined the affect that prenatal cigarette smoke exposure (PCSE) has on locomotor activity and cholinesterase activity in rats following either PCSE and/or ketamine treatment (acute subanesthetic doses of 5, 15, and 25 mg/kg) in adulthood and their potential relevance to schizophrenia [98]. They found that both PCSE and ketamine treatment induced increased locomotor activity and observed increased AChE and butyrylcholinesterase (BChE) activity in the brain and serum, respectively. These results suggest that higher levels of AChE and BChE result in the depletion of ACh at cholinergic receptors in the cerebral and peripheral areas that lead to subsequent declines in brain functions. Additionally, studies suggest that AChE overexpression disrupts the glutaminergic system, resulting in damage to synaptic structures and excitatory structures [107]. Thus, PCSE may lead to biochemical cholinergic effects that persist into adulthood, leading to dopaminergic upregulation and increased AChE activity that develop into symptoms of schizophrenia [98].

A follow-up study investigated the association between cigarette smoke during the prenatal period as a causative factor for obstetric abnormalities that lead to cognitive and behavioral changes associated with schizophrenia [99]. Pregnant rats were divided into non-PCSE and PCSE groups. Post-natal day 60 offspring received either saline or ketamine for 7 days and were then evaluated on various behavioral tasks. In the inhibitory avoidance task, the non-PCSE + ketamine, PCSE, and PCSE + ketamine groups all exhibited impairments in their long-term, short-term, and working memory. In the pre-pulse inhibition (PPI) test, PCSE + ketamine resulted in impaired PPI in 3 pre-pulse (PP) intensity groups as compared with the control groups, which is believed to be linked to a dysfunction in the sensorimotor gating mechanism. The non-PCSE + ketamine and PCSE + ketamine groups also exhibited increased locomotor activity which is likely resultant from an NMDA receptor blockade. Their biochemical analysis also revealed increased AChE activity in brain structures that was exacerbated in the PCSE + ketamine group [99]. Lastly, the PCSE + ketamine group exhibited a decreased latency in the social interaction test as compared with the non-PCSE + ketamine group, indicating that cigarette exposure may decrease the social deficits that are generated by ketamine. Overall, these results indicate that PNE may affect not only behavioral outcomes, but also the development of cholinergic structures in a manner that is like schizophrenia in humans.

### Autism spectrum disorder (ASD)

Autism spectrum disorder (ASD) is a neurodevelopmental disorder that’s characterized by the presence of restricted interests and repetitive behaviors and deficits in social communication [108]. According to the DSM-V, a child must have persistent deficits in each of three areas of interaction and social communication and at least two of four types of restrictive, repetitive behaviors [20]. The Centers for Disease Control and Prevention (CDC) estimates that approximately 1.68% of US children aged 8 years are diagnosed with ASD and the World Health Organization (WHO) estimates that the international prevalence of ASD is approximately 0.76% [109]. ASD is highly genetically heterogeneous and may be caused by both *de novo* and inheritable gene variations [110, 111]. ASD is understood to result from a complex interaction between genetics and the environment with heritability estimates currently ranging from 40% to 80% [112, 113]. Epidemiological investigations and extensive genetic studies have begun to elucidate the environmental factors that might contribute towards one’s risk and have revealed hundreds of genes linked to autism [110]. *In utero* exposure to nicotine has deleterious effects on prenatal neurodevelopment [114, 115]. As such, it is plausible to hypothesize that maternal smoking or nicotine exposure during pregnancy may influence one’s risk for developing ASD [116]. Due to the heterogeneous nature of ASD, a review of the current literature is important to assess whether there is an association between prenatal tobacco/nicotine exposure and one’s risk for developing ASD.

#### Clinical research

Clinical studies addressing the association between maternal smoking during pregnancy and ASD risk in offspring have yielded varying outcomes.

A case-control study of 3,958 ASD cases and 38,983 controls nested in a large register-based cohort in Sweden, for example, found that maternal smoking was associated with higher odds of any ASD (OR = 1.10, 95% CI: 1.01, 1.20), compared with no smoking [117]. A dose-response trend was also present with increasing ORs observed for higher levels of smoking (1-9 cigarettes daily: OR = 1.05, 95% CI: 0.95, 1.17; ≥ 10 cigarettes daily: OR = 1.18, 95% CI: 1.04, 1.33). These associations remained significant after adjusting for birth year, sex, adjusted maternal age, paternal age, and parity but lost significance when also adjusting for maternal/paternal education, maternal/paternal occupational class, family, income, and maternal origin of birth [117]. These results indicate that the apparent relationship of maternal smoking and ASD may be attributed to sociodemographic covariates.

A meta-analysis also investigated whether an association exists between PNE and ASD risk in offspring using a random-effects model to combine results in individual studies (6 cohort studies and 9 case-control studies; *n* = 17,890 ASD cases; *n* = 1,810,258 participants included in analysis) [118]. The results of their study indicated a pooled OR of 1.02 (95% CI: 0.93–1.13) when comparing offspring of mothers who smoked during pregnancy versus those that did not smoke during pregnancy. Thus, the result of their study found that there was no significant association between PNE and one’s risk for developing ASD [118]. However, the researchers noted that the study had several limitations to be considered including that the meta-analysis was based on observational studies, that PNE had the potential to be misclassified due to most of the studies being based on self-reported data, and that there was high statistical heterogeneity across the studies.

Another meta-analysis of 15 studies that addressed the risk for ASD in offspring exposed to maternal prenatal found that there was no evidence for an association (OR 1.02, 95% CI 0.93–1.12 for pooled analysis of all studies) [116]. When analyzing the included studies, they found that the strongest positive associations were reported when both the exposure and outcome were based on self-report, signaling recall bias. They also found that lower quality studies—ranked using the Newcastle-Ottawa Scale—tended to report positive associations and had greater between-study variance (τ^2^ = 0.2) while higher quality studies tended to report null findings and had lower between-study variance (τ^2^ = 0.01) [116]. Another meta-analysis examined 15 observational studies (6 cohort and 9 case-control) including 17,890 ASD cases and 1,810,258 controls/participants to explore whether an association exist between maternal smoking during pregnancy and ASD risk in offspring [118]. Their analysis found that maternal smoking during pregnancy was not associated with ASD risk in offspring (pooled OR = 1.02, 95% CI: 0.93–1.13) after adjusting for confounders [118].

In a recent meta-analysis published in *Nature* by Jung et al., the researchers utilized a novel approach of investigating population-level smoking metrics as a moderator between maternal smoking and ASD in offspring [119]. Analyzing 22 observational studies that comprised 795,632 cases and 1,829,256 control participants, their random-effects model found no significant association between maternal smoking during pregnancy and ASD in offspring (pooled OR = 1.16, 95% CI: 0.97–1.40). However, when they performed sub-group analysis, they found that smoking prevalence in males in the country of each study had a significant influence on ASD risk after maternal smoking (z = 2.33, *p* < 0.05). These results indicate that male smoking prevalence (found to correlate with secondhand smoke exposure) may have a significant impact on maternal smoking and ASD risk. In their discussion, however, the authors note the possibility of their findings reflecting the role of postnatal or paternal nicotine exposure as opposed to *in utero* or maternal nicotine exposure [119].

To account for the bias associated with self-reports of PNE, another study measured cotinine in stored second trimester maternal serum for 499 controls and 498 ASD cases born in California between 2011 and 2012 [120]. In addition to measuring cotinine levels, the researchers also obtained self-reported maternal cigarette smoking information during and immediately prior to pregnancy and covariate data from birth records. The results of their study found no association between cotinine concentrations and odds for developing ASD among children of non-smokers (aOR: 0.93 [95% CI: 0.69, 1.25] per ng/mL). Moreover, they found no associated between self-reported smoking (aOR:1.64 [95% CI: 0.65, 4.16]) or cotinine-defined smoking ( ≥ 3.08 ng/mL vs. < 3.08 ng/mL; aOR: 0.73 [95% CI: 0.35, 1.54]) and ASD [120].

Lastly, a population-based cohort and sibling-comparison design study conducted in California also examined the associations between maternal prenatal smoking and ASD in offspring [121]. They estimated the odds ratio for ASD with and without intellectual disability using California birth records (*n* = 2,015,104) that included information on maternal smoking, pregnancy, and demographic factors and ASD cases (*n* = 11,722) that were identified through the California Department of Developmental Services records [121]. In the full cohort, the aOR for ASD and maternal smoking 3 months before/during pregnancy compared with nonsmoking was 1.15 (95% CI: 1.04, 1.26) and was found to be similar between the cases with (OR = 1.12, 95% CI: 0.84, 1.49) and without intellectual disability (OR = 1.15, 95% CI: 1.04, 1.27) [121]. Mothers that smoked heavily during pregnancy ( ≥ 20 cigarettes/day in any trimester) were found to have an OR of 1.55 (95% CI: 1.21, 1.98) which indicates a dose-response relationship between maternal prenatal smoking and offspring risk for ASD [121]. These results differ from the previously discussed studies and indicates that further research must be conducted to establish whether a dose-response relationship exists between maternal prenatal smoking and ASD when controlling for demographic variables and other confounds.

#### Preclinical research

Due to the multifactorial nature of ASD, studies associating PNE with the development of autism are limited and more pre-clinical literature is necessary to determine whether a link between PNE and ASD are seen within animal models.

### Anxiety

Anxiety disorders are characterized by chronic and persistent excessive worrying that is typically accompanied by other nonspecific physical and psychological [122]. They are associated with unrealistic, persistent, and excessive worry about everyday things and a constant feeling of being overwhelmed [123]. The DSM-V classification of generalized anxiety disorders includes excessive anxiety and worry, occurring more days than not for at least 6 months with the anxiety, a difficulty controlling the worry, and the worry being associated with three or more of the following symptoms more days than not for at least 6 months: (1) restlessness or feeling keyed up or on edge, (2) irritability, (3) muscle tension, (4) sleep disturbance, (5) being easily fatigued, and (6) difficulty concentrating or mind going blank [20]. The symptoms must also cause clinically significant distress or impairment, must not be due attributable to the physiological effects of a substance or other medical condition, and must not be better explained by another mental disorder [20].

According to recent data, up to a 33.7% of the population are affected by an anxiety disorder over their lifetime with those suffering from anxiety at a significantly increased risk of other comorbid diseases such as depression, insomnia, and coronary artery disease [124]. Research suggests that anxiety disorders aggregate in families with first-degree relatives of patients with anxiety at a four to six times higher risk of developing an anxiety disorder as compared with relatives of healthy control subjects and twin studies estimating a heritability between 30% and 50% [125]. Segregation analyses implicate anxiety as a polygenic disease with a complex genetic inheritance pattern comprised of multiple susceptibility genes [126].

#### Clinical research

In child psychology and psychiatry, a well-known distinction has been made between “externalizing” and “internalizing” disorders [127]. Externalizing behaviors are displayed outwardly and include one’s behaviors towards their physical environment while internalizing behaviors are directed inwards and represent a child’s emotional and psychological state [127]. PNE has been consistently associated with symptoms of externalizing disorders such as hyperactivity but has been less clearly associated with internalizing disorders such as anxiety and depression [128]. Therefore, the present review presents a variety of clinical studies that seek to determine the relationship between PNE and internalizing behaviors such as anxiety.

A child cohort study assessed whether there was an association between PNE and internalizing behaviors in offspring (*n* = 90,040 mother-child pairs) [129]. The study assessed the status and frequency of smoking twice per pregnancy and maternal reports of their child’s internalizing behaviors using questions from the Childhood Behavior Checklist (CBCL) at 18 months, 36 months, and 5 years. They observed a dose-response relationship between frequency of PNE and childhood internalizing behaviors at 18 months and found that smoking 20+ cigarettes per daily conferred a much larger effect (B = 0.56, SE = 0.19, *p* < 0.01) than smoking 10–19 cigarettes (B = 0.14, SE = 0.04, *p* < 0.001), or 1–9 cigarettes (B = 0.1, SE = 0.20, *p* < 0.001) when compared with non-smoking controls. At 36 months and 5 years of age, however, no statistically significant differences were found after adjusting for covariates. These results indicate that a dose-response relationship between PNE and childhood internalizing behaviors (anxiety and depression) may be present at certain developmental stages [129].

A longitudinal prospective cohort study (*n* = 2,900 pregnancies) also assessed the influence of PNE on child and adolescent behavior development and found that children of light smokers (1–10 cigarettes daily) who quit smoking by 18 weeks’ gestation had significantly lower CBCL total z-scores than offspring of heavy smokers (11+ cigarettes daily) who quit and of offspring whose mothers continued smoking during pregnancy [130]. The result of their study indicates that a dose-response relationship may exist between PNE and an increase in internalizing symptoms that are associated with emotional disorders such as anxiety and depression. Their research also found that symptoms of internalizing may be ameliorated in offspring of light smokers that abstained from smoking after 18 weeks’ gestation [130].

An observational study and genome-wide gene-environment interaction study used subjects from the United Kingdom Biobank cohort (*n* = 371,903–432,881) to test the association between PNE and anxiety/depression in offspring [131]. Their analysis showed that PNE was significantly associated with anxiety and depression in offspring (*p* < 0.0001) as compared with non-exposed controls. Environment-gene interaction analysis also found a genome-wide significant SNP and several SNPs located in the UNC80 gene, a gene that produces a protein component of the sodium-ion channel complex (NALCN). Although there is no direct relationship between NALCN and anxiety, previous studies suggest that NALCN might be involved in the pathway between GABA receptors and anxiety and have been associated with neurodevelopmental changes that are associated with several anxiety-related risk factors [131–133].

#### Preclinical research

Due to differences in cognitive abilities between humans and rodents and the complex manifestations of psychiatric disorders, animal models of anxiety and stress disorders do not intend to replicate all features and symptoms of a specific anxiety disorder but rather generate a state of anxiety that may be related to the manifestation in humans [134]. In rodent models, conflict situations can be generated by opposite motivational states induced by approach-avoidance situations that either reflect an unconditioned general exploratory drive or responses that have previously been conditioned. Unconditioned animal models of anxiety include exploration-based models such as the elevated plus maze (EPM), elevated zero maze (EZM), light-dark box, novelty-suppressed feeding, and the social interaction test or predatory-based models such as the cat and rat exposure test [134]. Conditioned animal models of anxiety may include operant conflict tests such as the Geller-Seifter and Vogel conflict test and classical conditioning tests such as the place aversion, fear-potentiated startle, ultrasonic conditioning vocalization, and emotional conditioning responses tests. Thus, studies examining the effect of PNE on anxiety may use a combination of the designs to determine a rodent’s anxiety.

One study exposed pregnant rats to continuous infusions of nicotine (0.96 mg/kg/day or 2.0 mg/kg/day, freebase) and observed the effects on adult (P75) and adolescent (P30) animals [135]. They found that PNE had anxiogenic effects on adult rats in the EPM and blocked extinction learning in a fear conditioning paradigm. Their analysis of mRNA for the alpha-4, alpha-7, and beta-2 subunits of nAChRs also revealed lower expression of these subunits in the adult mPFC and hippocampus suggesting that PNE altered the developmental trajectory of the brain [135]. In another study, CD-1 dams were given injections of nicotine (1.5–2 mg/kg/day) throughout gestation and their offspring were administered a battery of behavioral tests at 6 months of age to assess sensorimotor integration and anxiety [136]. They found that PNE decreased directed exploration in the Suok test and that they had a decreased likelihood of jumping during the elevated platform test which both are indicative of increased anxiety. Moreover, they found that PNE exposed offspring exhibited increased anxiety and defects in sensorimotor integration that persisted into adulthood.

## Obesity and substance abuse

### Obesity

Obesity is defined by a body-mass index (BMI) of 30 kg/m^2^ or greater and has a prevalence of approximately 36.5% of men and 40.8% of women in the United States [137, 138]. Increasing body adiposity is associated with profound changes to one’s physiological functioning and increases one’s risk for the development of coronary heart disease (CHD), diabetes mellitus, stroke, respiratory complications such as obstructive sleep apnea, osteoarthritis, and certain forms of cancer [139]. Although environmental factors play a major role in the development of obesity, genome-wide association studies have found more than 50 genes associated with obesity [140]. Family, twin, and adoption studies have estimated the heritability of obesity to be between 40% and 70% with a strong genetic component underlying the interindividual variation in body weight [141]. As a multifactorial disease, obesogenic environmental factors such as physical activity, SES status, parent feeding behavior, alcohol consumption, and diet all interact with one’s genetics to promote the development of disease [142].

Clinical and animal studies have demonstrated an increased incidence of obesity in adolescents whose mothers smoked during pregnancy [11, 143, 144]. Although infants exposed to PN initially have low birth weights, a subsequent increase in obesity is observed later in life which likely reflects a greater preference for high caloric and palatable foods that activate the reward pathways [11]. As such, both preclinical and clinical literature have sought to study the association between PNE and neurodevelopmental changes to the brain reward pathways that underly one’s risk for obesity [11, 143, 144].

#### Clinical research

A variety of clinical studies have sought to determine the magnitude of the association between PNE and the risk for obesity in offspring. A systematic review and meta-analysis including 14 observation studies (*n* = 84,563 children), for example, found that children of mothers who smoked during pregnancy were at an elevated risk for being overweight at ages 3–33 years (OR = 1.50; 95% CI: 1.36, 1.65) when compared against children whose mothers did not smoke during pregnancy [145]. The researchers noted that the mothers who smoked during pregnancy tended to have characteristics that differed from non-smoking mothers in ways that may also predict one’s risk for childhood obesity including being more likely to have a lower income, being less likely to breastfeed, being less educated, and having children that were more inactive [146–148]. However, the researchers stated that the published studies they evaluated included different covariates with most accounting for fetal growth, measures of SES status, and fetal growth [145]. Moreover, when assessing the polled estimate from the unadjusted odds ratios (OR = 1.52; 95% CI: 1.36, 1.6), the risk was like the adjusted estimate and indicates that behavioral and social differences between smokers and nonsmokers are not likely to account for the observed differences in one’s risk for obesity [145].

Another systematic review and meta-analysis reviewed 39 studies of 236,687 children from Australia, Europe, North America, South America, and Asia to assess the relationship between PNE and being overweight between 2 and 18 years old [149]. The studies they reviewed included PNE rates ranging from 5.5% to 38.7% and that reported the prevalence of obesity from 2.6% to 17% and overweight from 6.3% to 32.1%. They found a pooled adjusted OR demonstrating an elevated odds of PNE and childhood obesity (OR = 1.55, 95% CI: 1.40–1.73; $I^{2}$ = 24%) and being overweight in childhood (OR = 1.37, 95% CI: 1.28–1.46; $I^{2}$ = 45%). These results demonstrate an increased risk of being overweight or obese during childhood in relation to PNE even when adjusting for potential confounders [149]. Similarly, another systematic review and meta-analysis of 17 observational studies examined the association between PNE and obesity of future offspring aged 3–33 years [150]. The meta-analysis found that PNE was significantly associated with an increased risk of obesity in offspring aged 3–33 years (OR: 1.64; 95% CI: 1.42–1.90) even after adjusting for publication bias and other confounders (aOR: 1.52; 95% CI: 136–1.70).

To determine whether a dose-response relationship exists between PNE and offspring overweight obesity, another meta-analysis of 26 studies (*n* = 238,340 mother-child pairs) examined the relationship between the number of cigarettes smoked during pregnancy and the risk for offspring overweight [151]. They found an overall increased ORs in offspring exposed to maternal smoking during pregnancy compared with offspring of mothers who did not smoke being overweight or obese (OR = 1.26; 95% CI = [1.22–1.29]). Assessing the dose-response relationship for both overweight and obesity in relation to the number of cigarettes smoked daily during pregnancy on a continuous scale, they found that the odds of a child being obese or overweight increased linearly up to 10–15 cigarettes per day, levelling out for doses higher than 15 cigarettes per day [151]. When adjusting for confounding variables, SES, maternal smoking, and paternal smoking, their analysis yielded similar results.

#### Preclinical research

To account for confounding variables, SES factors, and the effect of heritability on the relationship between PNE and risk for childhood obesity and overweight in offspring, studies have been conducted to examine the effect PNE in animal models.

One study in Wistar rats found that PNE resulted in an increased postnatal body weight and fat pad weight along with an increased amount of perivascular adipose tissue (PVAT) in the thoracic aorta and mesenteric arteries of PNE offspring [144]. Additionally, they found that PNE decreased the efficacy of the vascular relaxation response in offspring, raising concerns for blood pressure dysregulation and hypertension as the nicotine exposed animals age. Another study found that PNE adult male rat offspring had an increased inguinal subcutaneous fat mass and body weight and a decreased average cell area of adipocytes [152]. Although the PNE male pups did not consume more food than the controls, they had increased levels of adipogenic and lipogenic genes, increased levels of leptin mRNA, and higher serum insulin and lipid tests. When the male pups were tested at 26 weeks using the intraperitoneal glucose and insulin tolerance tests, they found that their glucose clearance was delayed and the area under the curve was higher for nicotine exposed offspring than the control group indicating the PNE increases the obesity susceptibility of adult male rats by altering early adipogenesis [152]. A study in Sprague Dawley rats exposed to PNE (3 mg/kg/day) also investigated the effect of gestational nicotine exposure on adipose tissue development and the early endocrine pancreas [153]. They found that PNE led to a decrease in endocrine pancreatic islet number at 7 days of life, led to an increase in epididymal white adipose tissue at weaning (postnatal day 21), and caused marked hypertrophy of adipocytes with increased gene expression of transcription factors involved in adipogenesis. They also found that PNE led to significant metabolic consequences such as increased cold intolerance, reduced physical activity, increased body weight and fat deposition, glucose intolerance combined with insulin resistance, and increased food efficiency on a high-fat diet. These results indicate that PNE causes early signs of dysregulation of adipose tissue and pancreatic development with an increased risk for excess body weight and obesity [153].

### Adolescent substance abuse

The incidence of substance use and abuse by adolescents is dependent on both genetic and environmental protective and risk factors [154]. Clinical and animal studies have demonstrated an increased incidence of adolescent substance abuse in adolescents whose mothers smoked during pregnancy [11, 143, 144]. Animal studies have also demonstrated that adolescents exposed to PN have an increased risk of dependence and misuse of drugs that activate the brain reward pathways [10]. As such, a review of both preclinical and clinical literature has sought to study the association between PNE and neurodevelopmental changes to the brain reward pathways that underly adolescent substance abuse [10, 11, 143, 144, 155].

#### Clinical research

A systematic review and meta-analysis of twenty-six cohort and one case-control study aimed to examine the association between prenatal tobacco exposure (PTE) and the risk of tobacco smoking and dependence in offspring [156]. The results of their study found elevated pooled risks of tobacco smoking initiation [RR = 2.08, (95% CI: 1.05–1.38)], tobacco dependence [RR = 1.50, (95% CI: 1.31–1.73], and current tobacco smoking [RR = 1.70, (95% CI: 1.48–1.95)] in offspring exposed to prenatal maternal tobacco exposure as compared with non-exposed offspring. Further, they found no association between paternal smoking during pregnancy and tobacco-smoking in offspring [156].

In a birth cohort study investigating whether there was a threshold effect for prenatal tobacco exposure (PTE) and adolescent risk for nicotine dependence (n- = 784), the researchers found that offspring of mothers that smoked 10 cigarettes per day during their first or third trimester were at a statistically elevated risk for nicotine dependence as compared to their non-exposed counterparts [10]. Their multivariate analysis also found that PTE remained significantly related to risk for offspring nicotine dependence after controlling for other covariates associated with adolescent cigarette use and maternal postnatal nicotine dependence [10]. Further, the results indicated that adolescents that were exposed to lower PTE had a similar ND risk as non-exposed adolescents [10].

Another prospective birth cohort study (*n* = 2,571) assessed whether maternal smoking during pregnancy would predict offspring nicotine disorder at 21 years [155]. Maternal smoking during pregnancy was assessed through maternal self-report, and offspring nicotine dependence or withdrawal was assessed using the Composite International Diagnostic Interview (CIDI) which assess nicotine withdrawal and dependence according to DSM-IV diagnostic criteria [155]. Their analysis found that offspring of mothers who smoked during pregnancy were more likely to experience nicotine dependence or withdrawal at 21 years than of offspring of mothers who never smoked (age adjusted odds ratio 1.53 (95% CI: 1.19–1.96) [155]. Further, their results remained significant even after adjusting for other factors such as birthweight, sex, and social disadvantage.

A birth cohort study by Cornelius et al. (2000) also sought to examine the relationship between PTE and early tobacco initiation in offspring [157]. The study included 589 ten-year-olds from low SES backgrounds that had been followed since gestation and included detailed information about prenatal and postnatal maternal substance use. Six percent of the children reported lifetime tobacco use with maternal smoking during pregnancy being significantly associated with an increased risk of early tobacco initiation. Their analysis found that prenatal tobacco exposure of >1/2 pack of cigarettes per day was associated with a relative risk of 5.50 (95% CI: 1.96–14.40). Peer tobacco use (RR = 11, 95% CI: 4.60–26.40), child delinquency (RR = 1.06, 95% CI: 1.01–1.12), the child’s depression (RR = 1.05, 95% CI 1.001–1.10), and maternal depression (RR = 1.05, 95% CI: 1.001–1.10) were all also significant predictors of tobacco experimentation during childhood. Despite these findings, further analysis confirmed that the association between PTE and childhood tobacco experimentation was not mediated by the child’s current environment or current maternal tobacco exposure [157]. A follow-up study of the prospective birth cohort followed the same group of participants at fourteen-years-old (*n* = 567) to determine whether there were trimester-specific effects of PTE on offspring smoking and other correlates of adolescent smoking [157]. They found that PTE in the third trimester significantly predicted offspring smoking when analyzed with demographic variables such as maternal and child psychological characteristics. However, when mother’s current smoking and peer smoking were included in their analysis, PTE was no longer found to be significant [157].

Studies examining sibling-pairs discordant for maternal smoking during pregnancy have also been conducted to control for familial vulnerabilities, either social or genetic, that predict risk of nicotine dependence [158, 159]. One study with sibling-pairs discordant for maternal smoking during pregnancy (*n* = 1,783), for example, found that PTE predicted progression from weekly smoking to nicotine dependence (OR = 1.4 [95% CI = 1.2, 1.8]), but did not predict progression to marijuana dependence in the exposed sibling [158]. Contrarily, another study examining sibling-pairs discordant for maternal smoking during pregnancy (*n* = 1,538) found that PTE had no apparent influence on tobacco dependence in adulthood [159].

#### Preclinical research

Animal models of addiction provide crucial information about the pathological mechanisms contributing towards the development of substance abuse in the absence of environmental confounds [160]. Animal models of abuse and addiction tend to focus on the ability of the drugs to directly control the animal’s behavior and to induce a rewarding effect that is not dependent on preexisting conditions [161]. Reports from drug abusers have also revealed that drug use was maintained not only by the rewarding properties but also their desire to alleviate craving or withdrawal symptoms [162]. As such, paradigms of drug addiction generally include features that include one’s vulnerability to drug abuse, how use transitions from controlled use to compulsive drug use, and how relapse to drug use occurs after a period of abstinence [161].

Preclinical studies in rodents have sought to determine whether a relationship exists between PNE and the offspring’s risk for altered reward pathway signaling and future adolescent substance abuse. One study in Lewis rats, for example, examined the effects of chronic gestational exposure to nicotine on the NA dopamine response to acute nicotine during adolescence [163]. They found that PNE diminished the adolescent NA dopamine response to nicotine administration and attenuated the NA dopamine release to a maximally stimulative dose of nicotine. Another study in rats evaluated the neurotoxicant effects of prenatal and adolescent nicotine exposure and found the PNE administration produced cellular alternations including net cell losses in the midbrain, and regional enlargement of the hippocampus [164]. Interestingly, they found that animals exposed to nicotine prenatally and given nicotine during adolescence had worse outcomes and greater neurotoxicity as compared to controls, indicating the neurotoxicant actions may contribute towards the association between PNE and subsequent smoking in offspring. A study in rats also investigated the influence of gestational nicotine on the offspring’s sensitivity to natural and drug rewards [11]. They examined PNE male adolescents on postnatal day 32 for operant responding using either sucrose pellets of i.v. cocaine (200 or 500 ug/kg) and addressed cocaine-induced stereotypy and c-fos mRNA expression in the cortex and striatum. They found that PNE induced complex changes in the offspring’s reward circuitry with the adolescent offspring not administering cocaine at low doses but exhibiting significantly greater cocaine intake and c-fos mRNA expression in the NA at higher doses than in the control group [11]. In contrast to the PNE rats, the control rats showed significantly greater drug-induced stereotypy at both cocaine doses.

Another study showed that sustained exposure to low doses of nicotine (3 mg/kg/day) during fetal development produced substantial changes in the developing corticostriatal dopamine system including both sex-dependent and sex-independent effects on the prefrontal dopamine systems [165]. PNE was found to alter norepinephrine transporter (NET) binding expression in both sexes and Catechol-O-methyl transferase (COMT)-dependent dopamine turnover in males. D2-like receptors and G-protein coupling in the ventral striatum was also enhanced in combination with altered roles of D2 and D3 receptors in cocaine-induced behaviors. These results demonstrate that low-dose PNE alters the development of the corticosteroid dopamine system in a manner that may underlie clinical deficits seen in adolescents such as increased incidence of substance abuse. Myelination defects in the CNS have also been implicated in various psychological disorders, including one’s risk for drug addiction [165]. One study, for example, treated Sprague Dawley rats treated with gestational nicotine (3 mg/kg/day) and found that PNE offspring (P35/P36) had altered myelin expression of myelin-related transcription and trophic factors that produced alterations in myelin gene expression and oligodendrocyte functioning [165].

Animal models have also demonstrated that PNE alters the development of the mesocorticolimbic system which is important for goal-directed behavior and its ability to modulate neural processes that contribute to drug abuse [166]. One study exposed rat dams to gestational nicotine (0.05 mg/kg/injection 3x/day on gestational days 8–21) and then exposed PNE offspring (P90) to 10 once daily injections of methamphetamine (0.3 mg/kg) to induce locomotor sensitization [167]. They found that PNE rats treated with methamphetamine showed significant enhancements in motor behavior, conditioned hyperactivity, and exhibited greater conditioned response of hyperactivity as compared to saline controls. PNE and methamphetamine administration also produced changes in BDNF protein levels throughout the mesocorticolimbic system [167]. These results suggest that PNE offspring have greater susceptibility to learn from drug-related conditional stimuli and produce alterations in neuronal plasticity that contribute towards drug abuse vulnerability.

## Electronic cigarettes (E-Cigs) and nicotine replacement therapy (NRT)

Although the prevalence of tobacco use has declined, other methods of nicotine consumption, such as electronic nicotine delivery systems and nicotine replacement therapies (NRTs), have become increasingly popular [168]. The perception that e-cigarettes are safer than tobacco cigarettes due to the presence of fewer hazardous chemicals may prompt pregnant women to begin use e-cigarettes as an alternative to tobacco cigarettes or other nicotine delivery devices [169, 170]. In one study, approximately 1.1% of women in the United States reported to have used e-cigarettes during the last 3 months of pregnancy [4]. Another study surveying pregnant women about their e-cigarette use (*n* = 7,434) reported that approximately 2.2% of pregnant women were current e-cigarette users and that 0.6% reported daily use [171]. Likewise, research has indicated that NRT (*n* = 339,875) was prescribed in 7.9% of pregnancies where mothers abstained from smoking tobacco cigarettes and in 1.7% of pregnancies where mothers utilized both NRT and tobacco cigarettes [172]. The growing percentage of women that utilize e-cigarettes during pregnancy and practitioners that prescribe NRT for smoking abstinence during pregnancy illustrate the importance of reviewing the literature on outcomes for expectant mothers and offspring.

At present, the effects of using e-cigarettes during pregnancy and early fetal development are largely unknown and few studies have addressed the association between e-cigarettes and neurobehavioral deficits [169]. Two recent epidemiological studies in humans have associated e-cigarette use with an increased risk for low birthweight and fetal growth restriction [173–175]. Studies in mice investigating the effects of e-cigarettes on different brain regions have found that e-cigarette exposure during pregnancy can alter the transcriptome of the frontal cortex (13–16 mg/mL nicotine), increase global DNA methylation within the brain (13–16 mg/mL nicotine), and dysregulate gene expression in the hippocampus (13 mg/mL nicotine) [176]. Mouse models of early life e-cigarette exposure have also demonstrated altered behavior patterns in adult offspring and reduced cognitive function (18–24 mg/mL nicotine) [177, 178]. Additionally, studies have reported that prenatal e-cigarette exposure (2.4% nicotine) in mice resulted in impaired locomotor, learning, and memory function in adolescent and adult offspring while disrupting the blood-brain barrier integrity [179].

Moreover, for both e-cigarette and NRT use during pregnancy, studies have found that plasma cotinine levels may approximate that of heavy tobacco cigarette smokers [180]. As such, the deleterious effects of nicotine on the developing brain are similar to those induced by tobacco cigarette exposure. Due to the serious consequences of PNE on the developing fetus, it is critical that healthcare practitioners and pregnant women are educated about the deleterious effects that both e-cigarettes and NRT have on offspring.

## Conclusion

Despite there being an established risk between prenatal maternal smoking and adverse outcomes, as of 2016 one in fourteen women (7.2%) in the United States reported to have smoked cigarettes during their pregnancy with the global prevalence of smoking during pregnancy estimated to be 1.7% [2, 3]. Due to the reinforcing effect that nicotine has on tobacco addiction behavior, pharmacologic interventions for tobacco cessation continue to be introduced including NRT’s in the form of gum, transdermal patches, oral inhalers, nasal sprays, and tablets as well as replacement through vaporized e-cigarettes [27]. Despite these efforts, research has demonstrated the PNE in any form has the potential to result in deleterious outcomes such as placental abruption, preterm birth, low birth weight and fetal growth restriction, stillbirth, and sudden infant death syndrome [5–7]. Moreover, clinical, and preclinical research has demonstrated that gestational nicotine exposure causes alterations to development in regions such as the PFC, VTA, NA, and caudate and may contribute towards the development of ADHD, schizophrenia, anxiety, obesity, and future adolescent substance abuse [17]. At present, there is inconclusive evidence to suggest whether PNE contributes towards the risk of ASD and more clinical and preclinical research is necessary to determine whether a true association exists.

In conclusion, the results of this review suggest the importance of ceasing smoking and any form of nicotine exposure during pregnancy to mitigate the risk of long-lasting complications on offspring.
